# Supplementary material for: Foliar mycoendophytome of an endemic plant of the Mediterranean biome (Myrtus communis) reveals the dominance of basidiomycete woody saprotrophs
Source: PeerJ. 2020 Dec 3;8:e10487. doi: 10.7717/peerj.10487 (PMC7719295; doi:10.7717/peerj.10487)
Supplement: Supplemental Information 1 [file peerj-08-10487-s001.docx]

Author: Rodrigo Bentes Kato

Contact: rbkato@gmail.com

01/10/2018

Pipeline for metagenomic ITS

This pipeline was elaborated and run on an Ubuntu 16.04.5 LTS system

Programs

- Vsearch v2.8.4

wget https://github.com/torognes/vsearch/archive/v2.8.4.tar.gz

- Blast v2.2.31+

sudo apt-get install ncbi-blast+

- Python

sudo apt-get install python

- Perl

sudo apt-get install perl

FASTA file processing

for R in *_R1.fastq ; do

vsearch --fastq_mergepairs ${R} --reverse ${R/_R1/_R2} --fastqout merged$

{arq}.fastq

**Merge paired-end sequence reads into one sequence.

vsearch --fastq_filter merged${arq}.fastq --fastq_maxee 0.5 --fastq_minlen

300 --eeout --fasta_width 0 --fastaout filtered${arq}.fasta

**Shorten and/or filter the sequences in the given FASTQ file and output the

remaining sequences to the FASTQ file specified

vsearch --derep_fulllength filtered${arq}.fasta --minuniquesize 2 --strand

plus --sizeout --uc dereplicated${arq}.uc --relabel sample_${arq}id_

--fasta_width 0 --output dereplicated${arq}.fasta

**Merge strictly identical sequences contained in filename. Identical sequences

are defined as having the same length and the same string of nucleotides (case

insensitive, T and U are considered the same).

end

Merge all samples

python3 catDerep_JF.py

Precluster before chimera detection

vsearch --cluster_size all_dereplicatedS.fasta --id 0.97 --strand plus --sizein

--sizeout --fasta_width 0 --uc precluster.uc --centroids precluster.fasta

**Clusterize the fasta sequences in filename, automatically perform a sorting

by decreasing sequence abundance beforehand.

Detect chimeras present in the fasta-formatted (denovo)

vsearch --uchime_denovo precluster.fasta --sizein --sizeout --fasta_width 0

--nonchimeras denovo_nonchimeras.fasta

**Without external references (i.e. de novo). Automatically sort the sequences

in filename by decreasing abundance beforehand (see the sorting section for

details). Multithreading is not supported.

Download UNITE database

https://unite.ut.ee/repository.php

Detect chimeras present in the fasta-formatted (reference)

vsearch --uchime_ref denovo_nonchimeras.fasta --db

uchime_reference_dataset_untrimmed_28.06.2017.fasta --sizein --sizeout

--fasta_width 0 --nonchimeras ref_nonchimeras.fasta

**Detect chimeras present in the fasta-formatted filename by comparing them

with reference sequences (option --db). Multithreading is supported.

Extract all non-chimeric, non-singleton sequences, dereplicated

perl map.pl all_dereplicatedS.fasta precluster.uc ref_nonchimeras.fasta >

nonchimeras_derep.fasta

Extract all non-chimeric, non-singleton sequences in each sample

perl map.pl dereplicatedS.fasta ll_dereplicatedS.uc nonchimeras_derep.fasta >

all_nonchimeras_derep.fasta

Clustering

vsearch --cluster_size all_nonchimeras_derep.fasta --id 0.97 --sizein --sizeout

--fasta_width 0 --relabel otu_ --uc otus.uc –centroids otus.fasta

**Clusterize the fasta sequences in filename, automatically perform a sorting

by decreasing sequence abundance beforehand.

Identification OTUs usando BLAST

blastn --db base -qcov_hsp_perc 90.0 -perc_identity 97.0 -query otus.fasta

-outfmt '6 qseqid stitle pident qcovhsp' -out taxonomy.blast

**BLASTn was used to compare the reads with the in house reference fungal

BLAST database. Only sequences with at least 97% similarity and at least 90%

coverage were identified. OTUs with ≥ 98,5% of similarity was assigned to

species level and similarities between 97 and 98,5% was assigned to genus

level.

Generating the Abundances Table

python abund.py

**We used a script written in python to parse the identification output and

generate a table in CSV (Comma-Separated Values) format

Some flags used:

--fastq_minlen~positive integer

With the --fastq_filter and --fastq_mergepairs commands, discard

sequences with less than the specified number of bases (default 1).

--fastq_maxee~real

With the --fastq_filter and --fastq_mergepairs commands, discard

sequences with more than the specified number of expected errors.

--fasta_width~positive integer

Fasta files produced by vsearch are wrapped (sequences are written

on lines of integer nucleotides, 80 by default). Set that value to 0 to

eliminate the wrapping.

--minuniquesize~positive integer

Discard sequences with an abundance value smaller than integer.

--relabel string

Please see the description of the same option under Chimera

detection for details.

--sizein

Take into account the abundance annotations present in the input

fasta file (search for the pattern "[>;]size=integer[;]" in sequence

headers).

--sizeout

Add abundance annotations to the output fasta file (add the pattern

";size=integer;" to sequence headers). If --sizein is specified, each

unique sequence receives a new abundance value corresponding to

its total abundance (sum of the abundances of its occurrences). If

--sizein is not specified, input abundances are set to 1, and each

unique sequence receives a new abundance value corresponding to

its number of occurrences in the input file.
