# Supplementary material for: Foliar mycoendophytome of an endemic plant of the Mediterranean biome (Myrtus communis) reveals the dominance of basidiomycete woody saprotrophs
Source: PeerJ. 2020 Dec 3;8:e10487. doi: 10.7717/peerj.10487 (PMC7719295; doi:10.7717/peerj.10487)
Supplement: Supplemental Information 2 — Conditional statements to classify the reads in taxa based on similarity. Reads greater than or equal to 97, gets genus; reads between 97 and 95, gets family; reads between 95 and 90, gets order; reads between 90 and 85, gets class; reads between 85 and 80, gets phylum; and reads less than or equal to 80 gets kingdom. [file peerj-08-10487-s002.pdf]

```
if (similarity >= 97):
    return genus                        #genus
elif (similarity < 97) and (similarity >= 95):
    return family                       #family
elif (similarity < 95) and (similarity >= 90):
    return order                        #order
elif (similarity < 90) and (similarity >= 85):
    return class                        #class
elif (similarity < 85) and (similarity >= 80):
    return phylo                        #phylum
elif (dado_id < 80):
    return kingdom                      #kingdom
end
```
