# Supplementary material for: Foliar mycoendophytome of an endemic plant of the Mediterranean biome (Myrtus communis) reveals the dominance of basidiomycete woody saprotrophs
Source: PeerJ. 2020 Dec 3;8:e10487. doi: 10.7717/peerj.10487 (PMC7719295; doi:10.7717/peerj.10487)
Supplement: Supplemental Information 3 [file peerj-08-10487-s003.docx]

##############################################################################################################

#Manuscript informations

##############################################################################################################

#Article title: Foliar mycoendophytome of an endemic plant of the Mediterranean biome (Myrtus communis) reveals the dominance of basidiomycotan woody saprothrophs

#Journal name: PeerJ

#Corresponding author: Aristóteles Góes-Neto

##############################################################################################################

rm(list=ls())

data<-read.csv2("/Users/alinevaz/Dropbox/Myrtus communis_endophytic fungi/ANTIGO Artigo Myrtus/Files - Aline/Analises/final_13_01_2020/myrtus_final_13_01_2020.csv", header=T)

data_org<-data[,-1]

rownames(data_org)<-c("T1","T2","T3","T4","T5","T6","T7","T8","T9","T10","T11")

##############################################################################################################

#Bipartite

require(bipartite)

library(bipartite)

##############################################################################################################

rm(list=ls())

data<-read.csv2("/Users/alinevaz/Dropbox/Myrtus communis_endophytic fungi/ANTIGO Artigo Myrtus/Files - Aline/Analises/final_13_01_2020/myrtus_final_13_01_2020.csv", header=T)

data_org<-data[,-1]

rownames(data_org)<-c("T1","T2","T3","T4","T5","T6","T7","T8","T9","T10","T11")

pdf(file = "/Users/alinevaz/Dropbox/Myrtus communis_endophytic fungi/ANTIGO Artigo Myrtus/Files - Aline/Analises/final_13_01_2020/Figure2.pdf" , width = 15, height =8)

par(mfrow=c(1,1))

par(mar=c(10,10,5,5))

plotweb(data_org, labsize = 1, col.interaction = "blue", text.rot = 90, y.width.low = 0.05, y.width.high = 0.05, ybig = 1.2)

dev.off()

pdf(file = "/Users/alinevaz/Dropbox/Myrtus communis_endophytic fungi/ANTIGO Artigo Myrtus/Files - Aline/Analises/final_13_01_2020/Figure3.pdf" , width = 15, height =12)

par(mfrow=c(1,1))

par(mar=c(10,10,5,5))

visweb(data_org, type = "diagonal", prednames = TRUE, preynames = TRUE, labsize = 2, plotsize = 12, textsize = 2)

dev.off()

networkLevel<- networklevel(data_org)# Table 2

speciesLevel<- specieslevel(data_org)# Table 3 and Table 4

data_bin<-(data_org>0)

shuffle.web(data_bin, N=2)

write.table(networkLevel,"/Users/alinevaz/Dropbox/Myrtus communis_endophytic fungi/ANTIGO Artigo Myrtus/Files - Aline/Analises/final_13_01_2020/NetworkLevel.xls", sep="\t", dec = ",")

write.table(speciesLevel$`higher level`,"/Users/alinevaz/Dropbox/Myrtus communis_endophytic fungi/ANTIGO Artigo Myrtus/Files - Aline/Analises/final_13_01_2020/SpeciesLevel_higher.xls", sep="\t", dec = ",")

write.table(speciesLevel$`lower level`,"/Users/alinevaz/Dropbox/Myrtus communis_endophytic fungi/ANTIGO Artigo Myrtus/Files - Aline/Analises/final_13_01_2020/SpeciesLevel_lower.xls", sep="\t", dec = ",")
